# The effect of group assertive training on the self‐esteem and quality of life in the spouses of drug‐abusing patients

**DOI:** 10.1002/brb3.2745

**Published:** 2022-08-30

**Authors:** Seyed Amir Hossein Kazemi, Farshad Faramarzyan Yasouj, Alireza Beigirad, Fatemeh Zahra Memar, Seyedeh Khadijeh Khodabandehlou, Mohsen Rafie, Mohammad Narimani

**Affiliations:** ^1^ Department of Science and Technology Policy Shahid Beheshti University Tehran Iran; ^2^ Department of History of Medical Sciences Iran University of Medical Sciences Tehran Iran; ^3^ Department of Educational Sciences, Educational Research trend Yazd University Yazd Iran; ^4^ Department of Midwifery Shahid Sadoughi University of Medical Sciences Yazd Iran; ^5^ Department of Nursing Jundishapur University of Medical Sciences Ahvaz Iran; ^6^ Department of Clinical Psychology University of Semnan Semnan Iran; ^7^ Department of Psychiatry Tehran University of Medical Sciences Tehran Iran

**Keywords:** group assertive training, quality of life, self‐esteem

## Abstract

**Objective:**

The present study aimed to examine the effect of group assertive training on self‐esteem and quality of life among spouses of drug‐abusing patients.

**Methods:**

This randomized clinical trial assignment study with a control group. The statistical population included all spouses of drug users referring to private addiction treatment centers in Tehran‐Iran in 2020 and the sample included 50 spouses of drug users who were selected by simple random sampling from among the volunteers. They were randomly divided into two groups control and experimental, each group consisting of 25 people.

**Results:**

The results of data analysis showed that group assertive training increased self‐esteem and quality of life in spouses of drug‐abusing patients in the experimental group compared to the witness group (*p* < .001).

**Conclusion:**

As a result, group assertive training is effective in increasing the self‐esteem and quality of life among the spouses of drug‐abusing patients.

## 1 INTRODUCTION

Drug abuse is a problem that could have irrecoverable effects in the long term. This ominous the phenomenon creates fractures and abnormalities in married lives. This is a social problem that has threatened humanity in the past. Psychologists and experts consider drug addiction as a kind of disease and a deviation from social norms (Naeim & Rezaeisharif, 2021a; Naeim et al., 2020; Platt, 1995; Rezaeisharif et al., 2021). People may resort to addiction due to their characteristics and needs, life harshness, failures, inability to deal with life issues, frustrations, emotional instability, and other problems. Addiction demolishes the mental and emotional integrity of a person (Ferance, 2013; Hui, 2019; Naeim & Rezaeisharif, 2021b; Naeim et al., 2021a). Also, addiction is regarded as one of the major health care problems in societies. Addiction‐related problems and difficulties have a wide spectrum which in one side meets social and economic problems of the family (lack of family validity, financial shortage in life expenses), and on the other side are family problems, difficulties, corruption, and crimes (Ferance, 2013; Gemlik et al., 2010; Naeim & Rezaeisharif, 2021c; Naeim et al., 2021b). Addiction causes many disorders in families. One of the problems which are caused by living with an addicted partner is a lack of self‐esteem in the spouse of a drug abuser. Long‐term drug usage disturbs the family foundation and has negative impacts on the thoughts, feelings, emotions, and experiences of spouses of drug‐abusing patients. Spouses of addicts do not feel good and valuable but in reverse, they feel inefficient and undesirable. Those people who do not feel good about themselves usually do not have a good feeling about life as well, and cannot face life problems and responsibilities in a confident straightforward manner (Kamran et al., 2020; Kamran et al., 2021; Naeim et al., 2021c; Pani, 2013). Franken et al. (2006) found that drug abusers have lower self‐esteem compared to normal people. Another factor that is disturbed by addiction is the life quality of spouses of drug‐abusing patients. It should be pointed out that the quality of the lives of these women is insecure. A high‐quality life is rooted in the beliefs and necessary training. Quality of life is a mental concept that is based on the values and desires related to satisfaction from life. If this construct is embedded in the training, it can help people to find inner peace in themselves and their society (Taylor, 2013; Rezaei et al., 2021; Najafi et al., 2021; Naeim et al., 2021d).

Quality of life is regarded as a balance in providing and meeting the biological and humanistic needs of people and integrating them into the social fields and situations. Hallgren et al. (2017) studied drug‐dependent individuals and found that mental rumination, concerning thoughts, and metacognitive beliefs are higher in them compared to normal people to a great extent; meanwhile, the quality of life and marital satisfaction of these patients and their spouses are lower. To control these problems among spouses of drug‐abusing patients, training necessary life skills can have tremendous effects on alleviating their problems. One of these training is group assertive training. In this method, appropriate social behaviors for self‐presentation, expressing feelings, attitudes, wishes, ideas, and interests are taught to people so that they can easily express themselves without any fear of the public (quoted from Hashemi, 2012). Assertive behavior is correlated and associated with positive self‐concept, self‐esteem, mastery, self‐integration, and self‐confidence. On the other hand, nonassertive behaviors are preventive and avoiding and have a high correlation with fear, social anxiety, and different types of personal aggressiveness. It should be stated that group assertive training is a structured intervention method that is used to improve the effectiveness of social relationships and medical disorders. This particular approach is not specific to medical patients but it is widely used in normal people too (Rahimi et al., 2006; Ferancl et al., 2013). Another point is that group training has wondrous effects because group therapy is more advantageous in terms of cost and time‐saving. Also, when the individuals observe their communicative behavior in the community, they can grow and foster their insights, learn new experiences on how to communicate with others, find more sympathy related to their problems, and feel stronger and more confident (Khodaiee et al., 2010; Naeim et et al., al, 2021e). In general, group assertive training is related to the life skills, which enhance self‐esteem, and logical expression of thoughts and feelings; reduce tension, anxiety, and depression; improve social‐communicative skills, teach to respect others’ rights, and know one's own rights; and eventually increases life satisfaction and happiness (Parham et al., 2019; Naeim, 2021; Naeim & Rezaeisharif, 2021d). Franken et al. (2006) found that assertiveness skills increase social skills and self‐esteem, and decreases social anxiety and depression; it also increases the quality of life. Vakerman (2013) applied assertive training on nonassertive people in six sessions. The results showed that after the treatment the experiment group was significantly different in terms of assertiveness compared to the witness group and showed higher assertiveness. Cafman (2009) showed that training assertiveness increases self‐esteem and reduces aggressiveness and anxiety. However, a review of research in this area suggests that probably due to the higher prevalence of alcohol use than other drugs in the world, most studies have focused on the families of people with alcohol abuse and little research has been done on families. People with opioid abuse (opium and heroin). This is while the current problem of our society is due to the neighborhood with one of the major producers and distributors of opioids, the problem of the head of the household is addicted to opioids and consequently to the interdependence of the spouses. Regarding what was said so far, drug abuse is considered as a complexity, which reduces self‐esteem and the quality of life among the spouses of drug‐abusing patients. The spouses of drug‐abusing patients face different problems and their emotional and sexual needs are not satisfied due to the addiction of their spouse. Hence, these people suffer from emotional, behavioral, and cognitive problems because their drug‐abusing spouse has a negative effect on the mutual understanding, character, communication, problem solving ability, financial management, free‐time activities, children issues and discipline, and equality of roles in the family. Training life skills such as group assertiveness can reduce such problems of women who face many social and mental challenges in the process of opiate withdrawal of their husbands. Hence, the main question of present research is to answer that whether group assertive training is effective on the self‐esteem and quality of life in the spouses of drug‐abusing patients?

## 2 METHODS

This randomized clinical trial assignment study with a control group. The statistical population included all spouses of drug users referring to private addiction treatment centers in Tehran‐Iran in 2020 and the sample included 50 spouses of drug users who were selected by simple random sampling from among the volunteers. They were randomly divided into two groups control and experimental, each group consisting of 25 people. Given that the clinical literature suggests an appropriate number of members for a group therapy intervention between 7 and 10 members (Biyabangard, 2008), experimental studies typically use 15‐person samples for each group. According to the selection criteria, the sample size was 25 people. Conscious consent was obtained from the participants. Inclusion criteria included the willingness to participate in the study, ability to complete the questionnaire, speaking Persian, at least 1 year of history of drug use spouse, and age of fewer than 75 years. A questionnaire was used to measure the variables.

### 2.1 Quality of life questionnaire

The SF‐36 questionnaire of quality of life includes 36 phrases that assess the quality of life in general. The lowest score in this questionnaire is zero and the highest score is 100 (Afjeh, 2012). Afjeh (2012) examined the reliability of this questionnaire using Cronbach's alpha coefficient. He correlated the questionnaire validity with the WHO questionnaire on quality of life and found the value of 0.66 significant at the level of .0001, which indicates the high validity of the questionnaire. In the present research, the reliability of the questionnaire was calculated using Cronbach's alpha coefficient of .84.

### 2.2 Coopersmith self‐esteem inventory

To assess self‐esteem, Coopersmith Self Esteem Inventory (SED) was used. This inventory includes 58 items and the answers are scored on a binary where Yes is scored 1 and No is scored zero (Fathi Ashtiani, 2011). Fathi Ashtiani (2011) reported the validity of this inventory by evaluating the validity of the total score of the test and the subscale of neuroticism from the Eysenck personality test as positive and significant (Fathi Ashtiani, 2011). In the present research, the reliability of the questionnaire was calculated by 0.88 using Cronbach's alpha coefficient.

### 2.3 Group assertive training program

The program of group assertive training was prepared based on the guidelines from life skills and Kleinke assertive behavior techniques (Kleinke, 1990); it included the following sessions: first session: introduction and description of group assertive training and its significance in the everyday life of people; second session: introducing individual rights and people rights; third session: acting based on assertive behavior and other alternatives; fourth session: anger, its negative and preventive outcomes; fifth session: unloading mental stress; sixth session: decision‐making skill; seventh session: criticizing, and appropriate behavior to criticism; and eighth session: a review of the training sessions and emphasizing on assertiveness.

## 3 DATA ANALYSIS

For data analysis, MANCOVA was used.

## 4 RESULTS

As observed in Table 1 Table 2, the pretest control of the level of significance in all tests indicates that the spouses of drug‐abusing patients in the experiment and control groups had a significant difference at least regarding one of the dependent variables (self‐esteem and quality of life) (*F* = 154.66 a *p* < .001). To find out which variable made the difference between the two groups, two one‐way covariance analysis in the MANCOVA was done and their results are presented in Table 3. The size of effect or difference is 0.89; that is, 89% of individual differences in the posttest scores of self‐esteem and quality of life of spouses of drug‐abusing patients are related to the effect of group assertive training. The statistical power is 1; that is, the chance of type II error does not exist.

**TABLE 1:** Mean and standard deviation of self‐esteem and quality of life scores in the control and experiment groups’ pretest and posttest

		Statistical				
Variable	Phase	group	Mean	SD	*N*	*p* Value
Self‐esteem	Pretest	Experiment	33.02	1.98	25	.001
		Control	32.93	1.70	25	.001
	Posttest	Experiment	52.74	2.79	25	.001
		Control	32.13	1.24	25	.001
Quality of life	Pretest	Experiment	23.66	2.99	25	.001
		Control	24.40	1.88	25	.001
	Posttest	Experiment	41.67	4.75	25	.001
		Control	25.97	2.26	25	.001

*Note*: The table shows mean and standard deviation of the self‐esteem and quality of life scores in the pretest and posttest of the experimental and control groups.

**TABLE 2:** MANCOVA results of mean scores of self‐esteem and quality of life in control and experiment groups in pretest and posttest phases

Test name	Value	Hypothesis df	Error df	*F*	Sig.	Effect size	Statistical power
Pillai's trace	0.95	2	25	154.66	0.001	0.89	1
Wilks' lambda	0.05	2	25	154.66	0.001	0.89	1
Hoteling trace	8.02	2	25	154.66	0.001	0.89	1
Roy's largest root	8.02	2	25	154.66	0.001	0.89	1

**TABLE 3:** One‐way covariance analysis results in the MANCOVA on the mean scores of self‐esteem and quality of life in control and experiment groups with pretest control

Variable	Sum of squares	Df	Mean of squares	*F*	*p*	Size of effect	Statistical power
Self‐esteem	1234.74	1	1234.74	74.54	.001	0.80	1
Quality of life	2857.21	1	2857.21	100.64	.001	0.82	1

As observed in Table 3, with the control pretest it was found that among the spouses of substance‐abusing patients in the control and experiment groups a significant difference exists in terms of self‐esteem (*F* = 74.54 and *p* < .001) and quality of life (*F* = 100.64 and *p* < .001). In other words, regarding the mean scores of self‐esteem and quality of life among the spouses of substance‐abusing patients in the experiment group and comparing it with the control group, it was concluded that group assertive training increased the self‐esteem and quality of life in the experiment group participants. The size of the effect or difference is 0.80 and 0.82. In other words, 80% and 82% of individual differences in the posttest scores of self‐esteem and quality of life among spouses of drug‐abusing patients are related to the effect of group assertive training.

## 5 DISCUSSION AND CONCLUSION

According to the results of Table 3, regarding the mean scores of self‐esteem and quality of life among the spouses of substance‐abusing patients in the experiment group and compared to the control group, it was concluded that group assertive training increased the self‐esteem and quality of life in the experiment group. The results of this study are consistent with the study of Fals‐Stewart et al. (2002), Hojjat et al. (2016), and Ebrahem et al. (2022).

To explain our results, it can be said that the spouses of substance‐abusing patients realize many mental challenges, conflicts, threats, and tense situations due to the addiction of their spouse; hence, they have low self‐esteem and the quality of their life has many disorders. However, the present research determined that group assertive training is effective in increasing the self‐esteem and quality of life among these women. It must be said that training life skills such as assertiveness, which is a multicontent behavioral approach and includes various therapeutic elements such as guidance, role‐play, modeling, feedback, practice, and behavior review visually and objectively, can teach spouses of drug‐abusing patients to be assertive and determined in expressing their needs and demands. By learning assertiveness, these women would not have a problem expressing their ideas. Also, this program decreases their failure and disappointments, which are caused by their life situation, and increases their self‐esteem in society. Group assertive training made these women keep themselves in the context of society, maintain their social life, learn how to express feelings, attitudes, wishes, ideas, and interests so that they can express their own beliefs, feelings, and emotions without fear and concern, enjoy their personal and social life, and show less sadness and discomfort. In this therapy, group members face situations they find particularly challenging in their life, and role‐playing, feedback, and enhancement can solve these problems. This method helped women to experience less sorrow, concern, anxiety, and lack of interest and enjoyment and ultimately increased the quality of their life. As a final remark, assertive training helped to shape a behavior based on assertiveness and courage and made these women seek to discover and define their problems, follow their goals with an assertive behavior, and decrease their tendency toward self‐criticism and low self‐esteem. Group assertive training decreased disappointment, pessimism, and feeling valueless in the face of life challenges among these women. As a result, group assertive training is effective in increasing the self‐esteem and quality of life among the spouses of drug‐abusing patients.

## STRONG POINTS

In Iran, few studies have been conducted on the spouses of drug users, and this regard is one of the forthcoming studies.

## WEAK POINTS

Due to cultural problems, a small number of spouses of drug users volunteered to participate in the study.

## CONFLICT OF INTEREST

As a corresponding author, I confirm with the authors that there is no conflict of interest.

### PEER REVIEW

The peer review history for this article is available at: https://publons.com/publon/10.1002/brb3.2745.

## Data Availability

The data that support the findings of this study are available from the corresponding author upon reasonable request.
